# Reduced ITPase activity and favorable IL28B genetic variant protect against ribavirin-induced anemia in interferon-free regimens

**DOI:** 10.1371/journal.pone.0198296

**Published:** 2018-05-31

**Authors:** Aparna Vasanthakumar, Justin W. Davis, Manal Abunimeh, Jonas Söderholm, Jiuhong Zha, Emily O. Dumas, Daniel E. Cohen, Jeffrey F. Waring, Martin Lagging

**Affiliations:** 1 AbbVie Inc., North Chicago, Illinois, United States of America; 2 Department of Infectious Diseases/Virology, Institute of Biomedicine, Sahlgrenska Academy, University of Gothenburg, Göteborg, Sweden; Nihon University School of Medicine, JAPAN

## Abstract

**Background:**

Genetic variants of inosine triphosphatase (ITPA) that confer reduced ITPase activity are associated with protection against ribavirin(RBV)-induced hemolytic anemia in peginterferon(IFN)/RBV-based treatment of hepatitis C virus (HCV). Patients with reduced ITPase activity showed improved treatment efficacy when treated with IFN/RBV. In addition, a genetic polymorphism near the IL28B gene is associated with an improved response to IFN/RBV treatment. RBV has been an important component of IFN-containing regimens, and is currently recommended in combination with several IFN-free regimens for treatment of harder to cure HCV infections.

**Aim:**

To evaluate whether genetic variations that reduce ITPase activity impact RBV-induced anemia in IFN-free/RBV regimens

**Methods:**

In this study, genetic analyses were conducted in the PEARL-IV trial to investigate the effect of activity-reducing ITPA variants as well as IL28B polymorphism on anemia, platelet (PLT) counts, and virologic response in HCV genotype1a-infected patients treated with the direct-acting antiviral (DAA) regimen of ombitasvir/paritaprevir/ritonavir and dasabuvir±RBV.

**Results:**

Reduction in ITPase activity and homozygosity for the IL28Brs12979860 CC genotype protected against RBV-induced anemia. In patients receiving RBV, reduced ITPase activity was associated with reduced plasma RBV concentration and higher PLT counts. ITPase activity had no impact on response to DAA treatment, viral kinetics, or baseline IP-10 levels.

**Conclusions:**

Our study demonstrates that genetics of ITPA and IL28B may help identify patients protected from RBV-induced anemia when treated with IFN-free regimens. Our work demonstrates for the first time that IL28B genetics may also have an impact on RBV-induced anemia. This may be of particular significance in patients with difficult-to-cure HCV infections, such as patients with decompensated cirrhosis where RBV-containing regimens likely will continue to be recommended.

## Introduction

Chronic hepatitis C virus (HCV) infection is of significant medical concern in the United States and worldwide, with a recent estimate of 71 million chronic infections and about 3–4 million new infections per year [1]. An estimated 20% of the chronically infected patients develop cirrhosis or liver cancer, necessitating liver transplantation within 20 years post-infection, therefore making early treatment of HCV infection crucial to preventing other disease manifestations [2,3].

First generation HCV treatments used interferons (IFNs) in combination with the guanosine analog ribavirin (RBV) [4]. However, response rates for these drugs varied from 30% to 90% depending on specific viral genotype and patient characteristics [5–7]. Direct acting antivirals (DAA) were approved in 2011 for use in combination with IFN and/or RBV and demonstrated higher response rates compared to earlier DAA-free regimens [8–11]. Additionally, newer IFN-free DAA regimens have further increased sustained virological response (SVR) rates, decreased the duration of treatment, and demonstrated particular effectiveness in populations with harder to cure infections such as patients with cirrhosis and those who have failed prior treatment. In some settings RBV has proved to be necessary to maximize SVR rates, therefore RBV is still recommended for use in combination with some DAA regimens for the treatment of specific patient populations [12,13]. In particular, RBV likely will remain important in the treatment of patients with decompensated cirrhosis, as the general use of HCV protease inhibitors is not recommend in these patients [14]. While the addition of RBV to DAA regimens improves efficacy in some patient populations, use of RBV is also associated with adverse events. One of these is hemolytic anemia likely due to the accumulation of RBV-triphosphate within the erythrocytes which has been hypothesized to result in oxidative stress and red blood cell lysis through the depletion of adenosine triphosphate (ATP) and guanosine triphosphate (GTP) [15,16].

Several pharmacogenetic variants have been shown to impact response to HCV therapy and the associated safety events. The single nucleotide variant rs12979860 (also known as interleukin 28B or IL28B ‘CC’ genotype) associates with a greater likelihood of achieving SVR in several pegylated (PEG)-IFN/RBV treatment studies [17–19]. However, the exact mechanism of the improved response has not been demonstrated. More recently, it has been reported that that the rs12979860 variant resides within the newly discovered interferon lambda 4 (IFNL4) gene, and may impact SVR via regulation of the expression of the IFNL4 gene [20–22]. A second gene that associates with HCV treatment is the inosine triphosphatase (ITPA) gene, which encodes an enzyme that catalyzes pyrophosphohydrolysis of ITP to inosine monophosphate (IMP) to protect against accumulation of non-canonical nucleotides that induce genomic instability [23]. Several polymorphisms have been identified in the ITPA gene, two of which are associated with reduced ITPase activity [24]. Reduced ITPase activity has been associated with protection against hemoglobin (Hb) decline after treatment with peg- IFN and RBV [24–26]. In addition, reduced ITPase activity is associated with a greater decline in platelet (PLT) counts. Both clinical phenotypes have been attributed to reduced depletion of ATP, since ITP may substitute for GTP in the cell, which prevents oxidative damage and subsequent erythrocyte membrane damage [27,28]. The current study was initiated to understand the effect of reduced ITPase activity on RBV-induced anemia and change in PLT counts in a patient population that received an IFN-free DAA regimen with or without RBV.

## Materials and methods

### Patients

**Fig 1:**
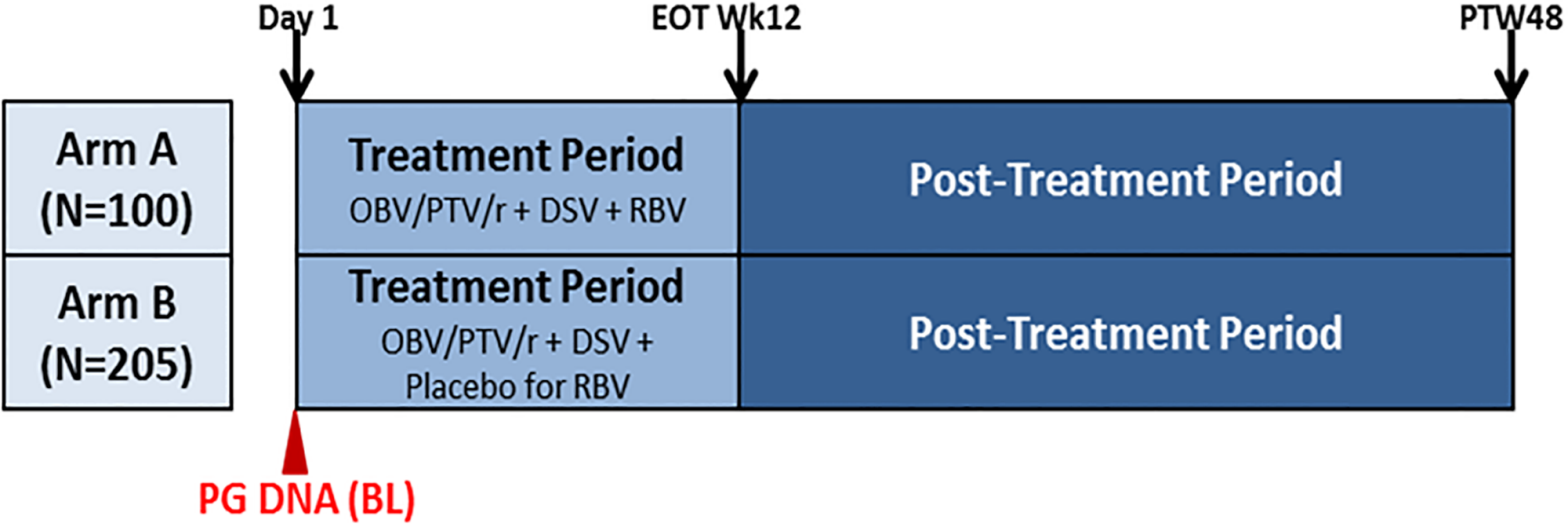
Study schematic for M14-002 (PEARL-IV). During treatment period subjects received 12 weeks of therapy of OBV/PTV/r (150/100/25 mg QD) + DSV (250 mg BID) + RBV (or placebo for RBV). In the Post-Treatment Period, all subjects administered at least one dose of study drugs were followed for 48 weeks to monitor for safety, HCV RNA, the emergence and/or persistence of resistant viral variants and assessment of patient-reported outcomes.

PEARL-IV (NCT01833533) was a randomized, double-blind, placebo-controlled trial to evaluate the efficacy and safety of the 3-DAA regimen consisting of ombitasvir/paritaprevir*/ritonavir (*identified by AbbVie and Enanta) and dasabuvir with or without RBV in treatment-naïve adults with genotype 1a chronic HCV Infection (Fig 1), as described elsewhere [12]. The study was conducted in accordance with the International Conference on Harmonization, applicable guidelines governing conduct of clinical studies, and ethical principles originating in the Declaration of Helsinki. The protocol and amendments were approved by an independent ethics committee or Institutional Review Board at each study site. Only samples from patients who consented to optional pharmacogenetic analysis were included in this study. The full list of IRB sites which approved the protocol for patient samples used in the pharmacogenetic analysis are listed in S1 Table. Only patients who identified as White were included in this analysis, since there were smaller numbers of patients (N = 1–29) for each non-White category (Black, Asian, Native American, Pacific Islander) (Table 1).

**Table 1:** Number of patients from PEARL-IV analyzed for pharmacogenetics.

	Total # Patients	# Patients with Pharmacogenetics Consent	# Patients Analyzed*
**Arm A** **(DAA+RBV)**	100	68	58
**Arm B** **(DAA+Placebo for RBV)**	205	157	131

*Patients who identified as Non-White (Black, Asian, Native American, Pacific Islander, or American Indian) were excluded from this analysis owing to small numbers in each category

### DNA isolation and processing

DNA was isolated from a 4-mL whole-blood sample collected into an EDTA-containing tube from each consenting subject during the screening period and stored at −20°C until DNA extraction. DNA was isolated from whole blood using Qiagen reagent kits (Qiagen Inc, Valencia, CA, USA) applied to an AutoGenprep 3000 (AutoGen Inc, Holliston, MA, USA) automated DNA extraction instrument.

### Genotyping

Targeted sequencing of the nucleotide polymorphisms rs12979860 (C>T), ITPA rs1127354 (C>A) and ITPA rs7270101 (A>C) was done using the pyrosequencing detection method (Qiagen Inc) according to the manufacturer's recommended protocol and using the primers listed in S2 Table. Conditions for Hardy-Weinberg equilibrium were met as outlined in S3 Table.

### Statistical methods

Regression analysis (General Linear Model [GLM]) was performed to determine whether ITPase activity altered Hb levels, RBV concentrations, or PLT counts with covariates that included rs12979860 genotype, age, and sex. All three-way interactions and lower order terms were considered in the model, and the best-fitting model was chosen based on the lowest AIC numbers (Akaike Information Criterion) to avoid overfitting. Residual plots and diagnostics were assessed to evaluate model fit. This approach allows for the possibility of determining which effects may be independent of the other effects (e.g., if the impact of rs12979860 genotype on Hb change is independent of ITPA level, then the rs12979860*ITPA interaction term would not be in the best-fitting model). All statistical calculations were done using SAS 9.4.

## Results

ITPase activity was estimated from the compound genotypes of rs1127354 and rs7270101 as previously described using biochemical analysis [27,28]. Eighty two percent of the patients tested had ITPase activity ≥ 60%, and approximately 4% of the patients had ITPase activity ≤10% (Table 2).

**Table 2:** Distribution of ITPA genotypes and corresponding ITPase functional activity in PEARL-IV.

rs1127354	rs7270101	Predicted ITPase activity	Distribution in PEARL-IV % (n)	
			-RBV 70% (157)	+RBV 30% (68)
Wild-type (CC)	Wild-type (AA)	100%	42% (93)	18% (41)
Wild-type (CC)	Heterozygote (AC)	60%	16% (36)	6% (14)
Wild-type (CC)	Homozygote (CC)	30%	0.9% (2)	0% (0)
Heterozygote (CA)	Wild-type (AA)	25%	8% (18)	5% (12)
Heterozygote (CA)	Heterozygote (AC)	10%	2% (5)	0.4% (1)
Homozygote (AA)	Wild-type (AA)	<5%	0.9% (2)	0% (0)

**Fig 2:**
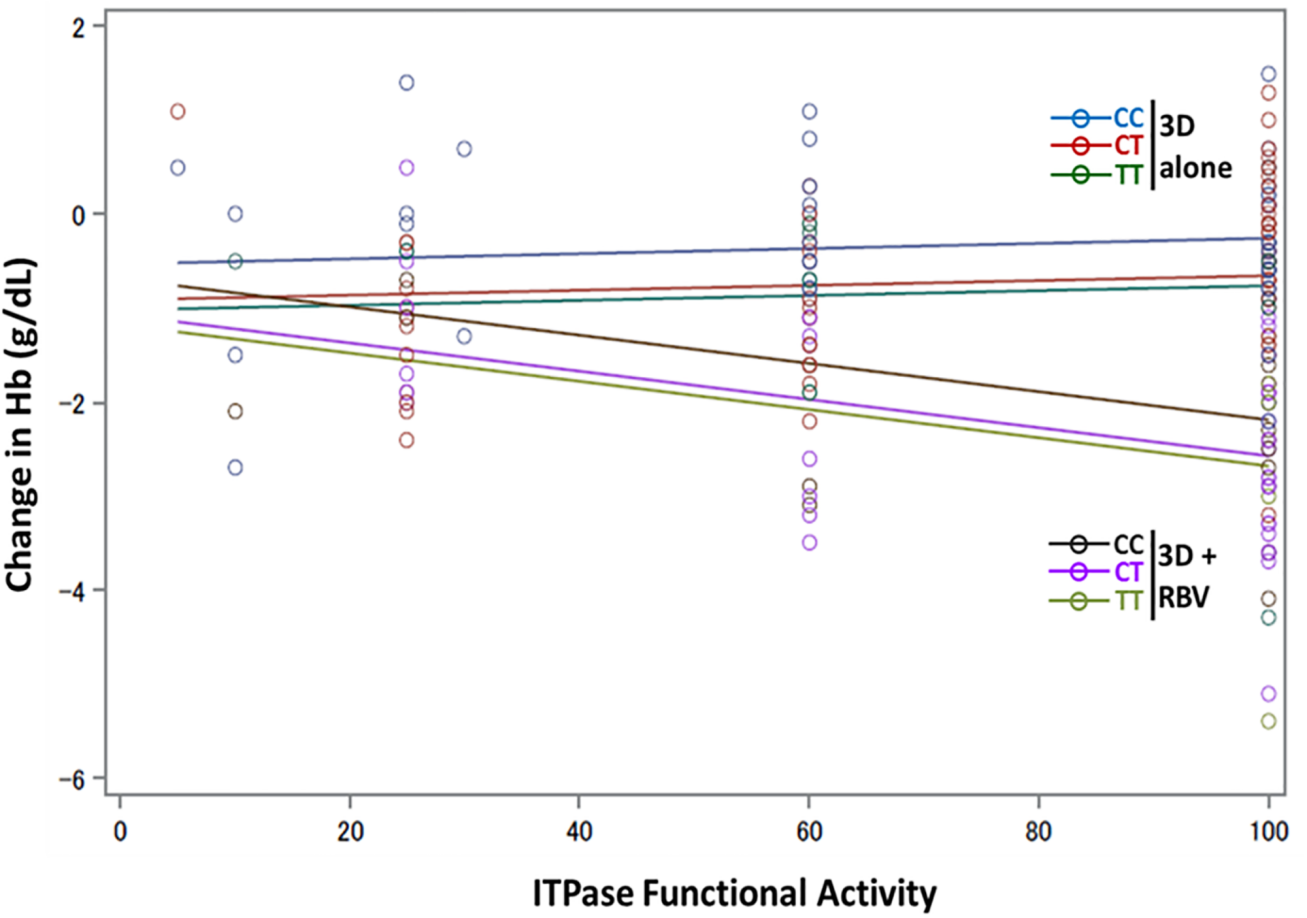
Low ITPase activity and the rs12979860 CC genotype protect against RBV-induced anemia. Hemoglobin (Hb) change from baseline (BL) to end of treatment (EOT) was calculated and regression modeling was done to illustrate the association of Hb changes with ITPAse functional activity and rs12979860 Genotype. The 2 arms are indicated, and are categorized by rs12979860 genotype. Line color indicates the fit for regression analysis: Blue- IFNL4 CC in placebo arm; Red- rs12979860 CT in placebo arm; Green- rs12979860 TT in placebo arm; Black- rs12979860 CC in RBV arm; Purple rs12979860 CT in RBV arm; Light Green- rs12979860 TT in RBV arm.

The change in Hb from baseline to end of treatment (EOT) was tested for associations with the derived ITPase activity. A significant reduction was observed in least squares mean of Hb levels at EOT in the DAA + RBV arm, which was significantly different from the DAA + placebo for RBV arm (S1 Fig). GLM in conjunction with the lowest AIC was used to select the final model, and a model that included arm, ITPase activity, and rs12979860 genotype was selected (R2 = 40.2%; p<0.0001) of all models evaluated (Table 3). ITPase activity was significantly associated with change in Hb in the DAA+RBV arm (Fig 2). Patients with low ITPase activity who received RBV were protected against anemia, whereas patients with high ITPase activity who received RBV developed anemia at a significantly higher rate (Fig 2; S4 Table). Additionally, there was a significant association between rs12979860 genotype and the change in Hb (Table 3, S2 Fig). This association was independent of whether the subject received RBV or not. Within each treatment arm, the presence of at least one unfavorable T allele for the rs12979860 polymorphism rendered the subject less protected against anemia relative to the favorable CC genotype (Fig 2). A genotype (2 df) genetic model term was used in all multivariate models. The rs12979860 SNP allele status was an independent predictor of Hb change (S2 Fig). Additionally, we did not find any significant differences in Hb changes between male and female patients (S3 Fig).

**Table 3:** GLM analysis for Hb change shows model is useful (p<0.0001, R2 = 40.2%).

**Source**	**Df**	**Sum of Squares**	**Mean Square**	**F value**	**Pr>F**
**Model**	5	119.68	23.94	24.47	<0.0001
**Error**	182	178.062	0.978		
**Corrected Total**	187	297.744			
**R_square**	**Coeff Var**		**Root MSE**	**HbChange Mean**	
**0.402**	-91.06526		0.9891	-1.08617	
**Source**	**Df**	**Type III SS**	**Mean Square**	**F-value**	**Pr>F**
**Arm**	1	0.1187	0.1187	0.12	0.7280
**ITPase**	1	5.3424	5.3424	5.46	0.0205
ITPase*Arm	1	10.9888	10.9888	11.23	0.0010
**rs12979860**	2	7.098	3.549	3.63	0.0285

**Fig 3:**
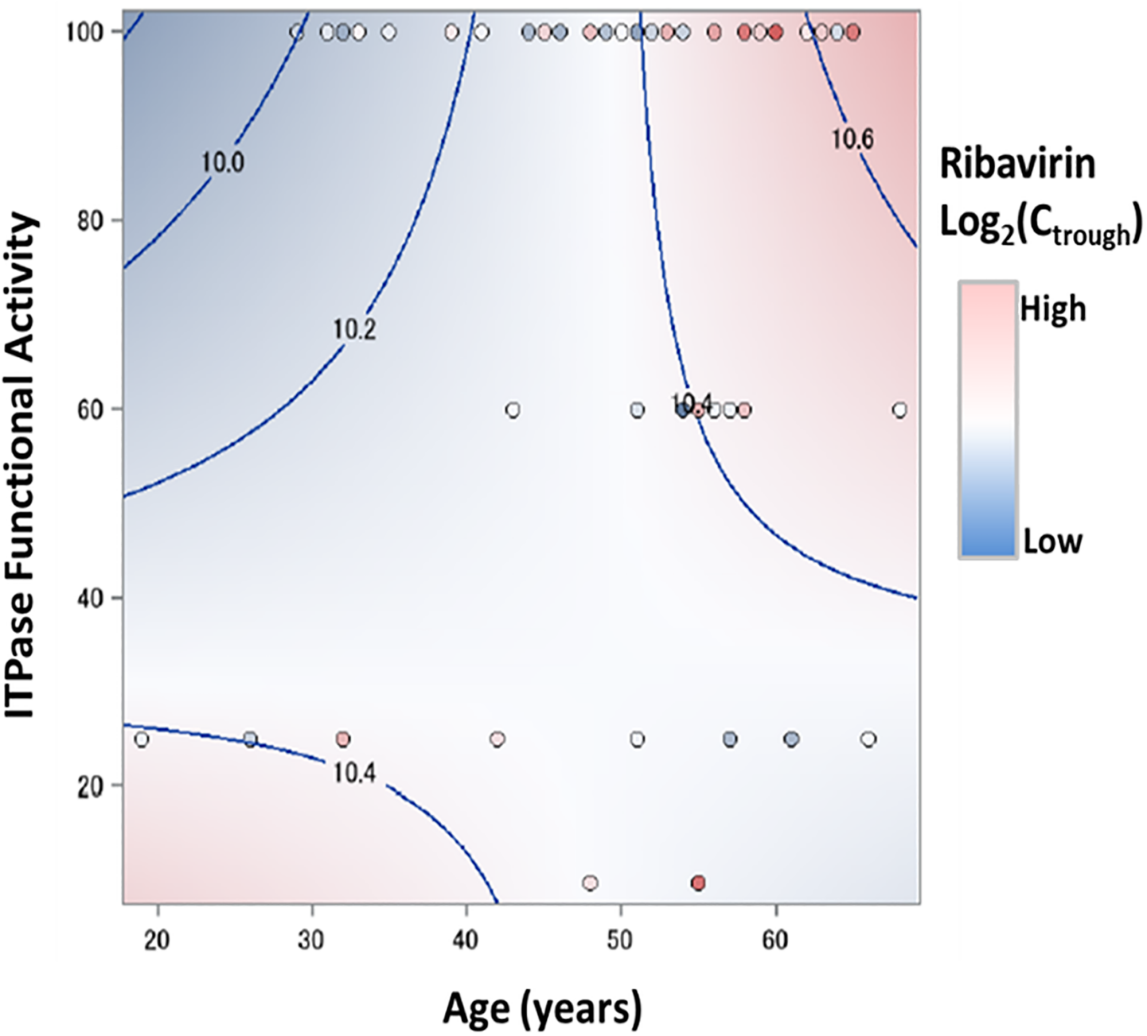
RBV log2(Ctrough) is dependent on ITPase activity and age. The contour fit plot for RBV log2(Ctrough) was generated using ITPase functional activity, RBV log2(Ctrough), and age of the individual.

Previous work had demonstrated that reduced ITPase activity may be associated with lower concentration of RBV in the plasma, which may contribute to protection against anemia [29]. We sought to examine the association of ITPase functional activity with plasma RBV concentration (measured by log2[Ctrough]) in the DAA+RBV arm). A GLM using age and ITPase functional activity as covariates showed a significant association (p = 0.0269; R2 = 15.3%) of ITPase functional activity with plasma RBV concentrations (Table 4). Our data indicate that RBV log2(Ctrough) is determined by an interaction of ITPase activity and age, and that reduced ITPase activity is associated with reduced RBV concentration (Fig 3). After controlling for covariates, we found no significant association of log2(Ctrough) with rs12979860 genotype at any level of ITPA functional activity (S4 Fig).

**Table 4:** GLM analysis of plasma RBV concentration in Arm A (DAA+RBV) shows the model is useful (p = 0.0269, R2 = 15.3%).

**Source**	**Df**	**Sum of Squares**	**Mean Square**	**F value**	**Pr>F**
**Model**	3	1.3088	0.436	3.30	0.0269
**Error**	55	7.265	0.132		
**Total**	58	8.574			
**R_square**	**Coeff Var**		**Root MSE**	**Log2(Ctrough) Mean**	
**0.153**	3.4963		0.3635	10.395	
**Source**	**Df**	**Type III SS**	**Mean Square**	**F-value**	**Pr>F**
**Age**	1	0.094	0.094	0.71	0.4023
**ITPase**	1	0.543	0.543	4.11	0.0474
Age*ITPase	1	0.579	0.579	4.38	0.0409
**Solution for Fixed Effects**					
**Effect**	**Estimate**	**Standard Error**	**DF**	**T Value**	**Pr>|t|**
**Intercept**	10.7672	0.4958	55	21.72	<0.0001
**ITPase**	-0.012994	0.006378	55	-2.03	0.0474
**Age**	-0.00840	0.009956	55	-0.84	0.4023
ITPase*Age	0.00026	0.000126	55	2.09	0.0409

**Fig 4:**
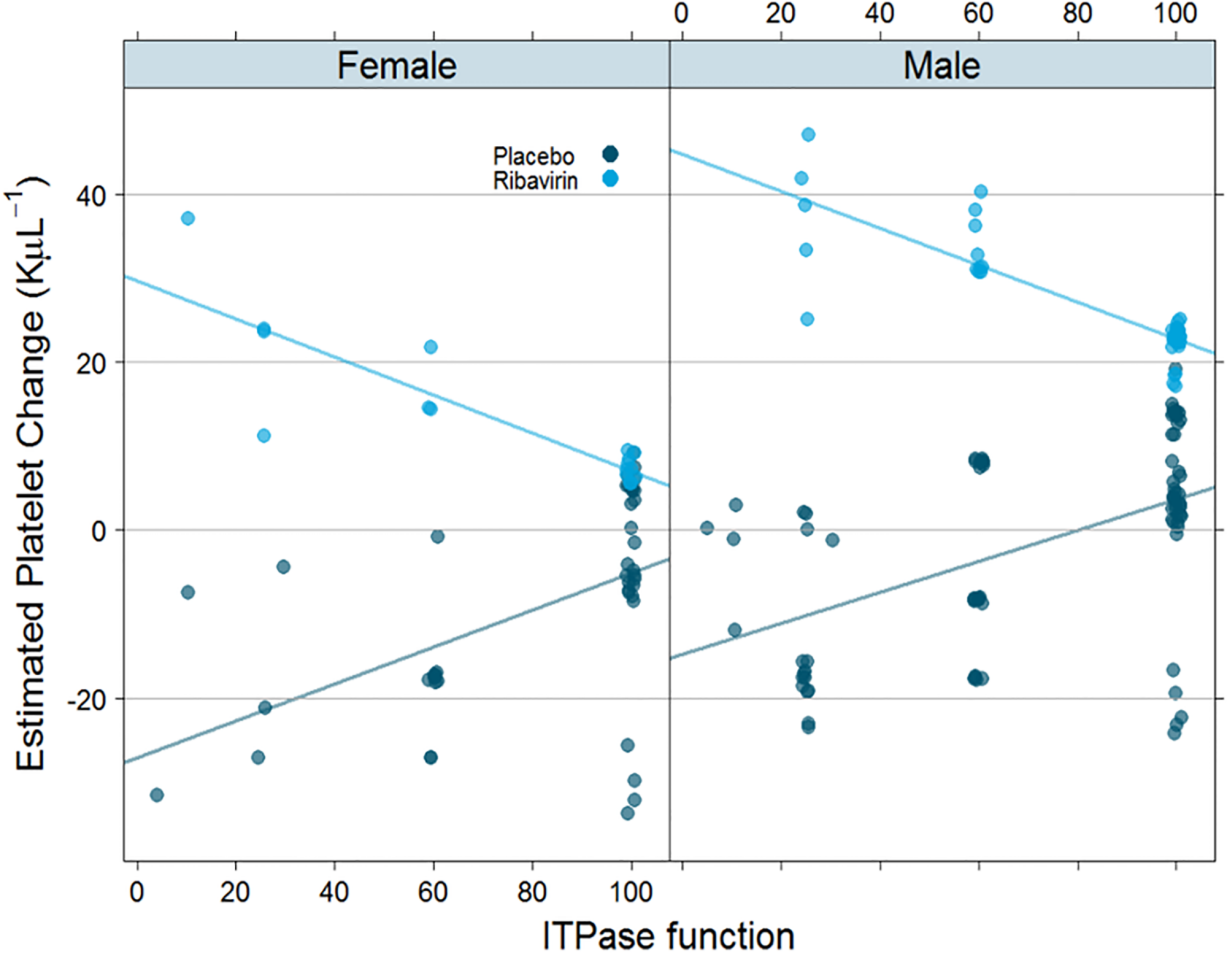
High ITPase activity correlates with reduction in PLT numbers in the presence of RBV. Estimated PLT change in female subjects (left) and male subjects (right) shows distribution as a function of ITPase activity. DAA + RBV arm is denoted by light blue dots and DAA + Placebo by dark blue dots.

To test whether reduction in ITPase activity associates with a decline in PLT counts, as has previously been demonstrated [30], we tested for linear associations between changes in PLT counts at EOT with ITPase activity. Model selection criteria indicated that several covariates should be included in the model, and the final model did demonstrate that was it useful with a significant (p = .0033, R2 = 16.7%) association of ITPase activity with PLT changes (Table 5). The interaction of ITPase activity with arm was significantly associated with reduction in PLT counts (Table 5; S4 Table). Both males and females demonstrated a reduction in PLT number that was associated with elevated ITPase activity (Fig 4). The distribution of absolute PLT change was not associated with rs12979860 genotype (S5 Fig). After controlling for all covariates, treatment arm (DAA with or without RBV) was associated with the differential changes in PLT counts, whereas rs12979860 genotype did not predict alterations in PLT counts at any level of ITPase functional activity (S6 Fig).

**Table 5:** GLM analysis of change in PLT counts shows best model is useful (p = .0033, R2 = 16.7%).

**Source**	**Df**	**Sum of Squares**	**Mean Square**	**F value**	**Pr>F**
**Model**	14	54700.2	3907.2	2.47	0.0033
**Error**	173	273894.6	1583.2		
**Corrected Total**	187	328594.9			
**R_square**	**Coeff Var**		**Root MSE**	**PLT Change Mean**	
**0.167**	988.168		39.79	4.0266	
**Source**	**Df**	**Type III SS**	**Mean Square**	**F-value**	**Pr>F**
**ITPase**	1	266.33	266.33	0.17	0.6822
**Arm**	1	5192.79	5192.79	3.28	0.0720
ITPase*Arm	1	7353.25	7353.25	4.64	0.0325
**rs12979860**	2	2485.27	1242.64	0.78	0.4578
ITPase* rs12979860	2	2521.016	1260.51	0.80	0.4527
Arm* rs12979860	2	2896.34	1448.17	0.91	0.4026
**Age**	1	638.71	638.71	0.4	0.5262
ITPase*Age	1	724.56	724.56	0.46	0.4996
Age*Arm	1	65.33	65.33	0.04	0.8393
**Sex**	1	5235.88	5235.88	3.31	0.0710
Arm*Sex	1	442.42	442.42	0.28	0.5977

Rate of response to treatment (SVR12) in PEARL-IV was 97% in Arm A (DAA+RBV) and 90% in Arm B (DAA+Placebo) within the intent-to-treat (ITT) population. We did not observe any associations of ITPase functional activity with the response rates in either arm (S5 Table). Additionally, reduction in ITPase activity had no association with viral kinetics, or baseline IP-10 levels.

## Discussion

We demonstrate in this study that rs12979860 genotype and ITPase activity regulate RBV-induced anemia in HCV-infected patients treated with an IFN-free DAA regimen. The effect of ITPA genotype on RBV-induced anemia has been shown in several studies both within IFN-containing and IFN-free regimens [24,25,29–36]. In some of these studies, patients carrying the polymorphisms that predicted low ITPase functional activity not only had a delay in onset of anemia, but required a smaller reduction in RBV dose [26,30]. In this study, we demonstrate a similar protection against RBV-induced anemia in patients with ITPase functional deficiency. Two other recent papers have demonstrated this modulation of severity of anemia in RBV-containing IFN-free regimens [35,36]. In addition, our work reveals a significant novel association of the rs12979860 genotype with RBV-induced anemia. The CC genotype at this locus predicts a lower frequency of RBV-induced anemia relative to patients carrying at least one of the T alleles. Although the precise molecular mechanism linking the rs12979860 SNP to IFN/RBV response has not been identified, its impact on immune function in response to HCV infection has been demonstrated. Additionally, there was an association of *IFNL* genotype with systemic iron balance, which may play a role in impacting peripheral iron status[37]. A recent paper which described clinical trial simulations in a mathematical model of RBV-induced anemia in HCV patients suggested, in contrast to our observations, that the non-CC rs12979860 patients had the least severe anemia [38]. This difference may be a result of the type of antiviral therapy: the data used for the simulations in the Wu paper were from IFN-containing RBV trials whereas ours was an IFN-free DAA trial. The protective effect of the ITPA polymorphism on RBV-induced anemia is not yet well characterized. It is hypothesized that RBV induces a decrease in erythrocyte ATP levels, which hinders oxidative metabolism within the cell leading to widespread hemolysis [16]. In the case of ITPase functional deficiency, it has been speculated that the accumulation of intracellular ITP allows ATP biosynthesis to continue by replacing the GTP within the cell for metabolic processes [39].

In contrast to its effect on anemia, the impact of ITPase functional deficiency on response to RBV-containing treatment has yielded conflicting evidence. Several reports have shown no association of ITPase functional activity with treatment response [33,34,40,41]. However, other studies in patients treated with IFN/RBV demonstrated associations of ITPase functional activity with treatment response. In the NORDynamic trial, patients receiving peg-IFN-2α and RBV (800 mg) had an improved response when they were deficient for ITPase functional activity [29]. This was mediated by a reduced risk of relapse in this patient population. In a Japanese phase 2 study, the genotype at rs1127354 (ITPA) was found to be associated with improved treatment outcome, provided there was adherence to RBV [42]. In PEARL-IV, we did not see any association of SVR with ITPase functional activity, possibly due to the high efficacy of the new IFN-free DAA treatments. Additionally, non-White patients were excluded from our analysis due to lack of sufficient numbers in each of the groups (Table 1) and most virologic failures in both the arms of PEARL-IV were among non-White patients [43].

Higher rates of anemia have previously been associated with increased RBV exposure [29,44]. Our current analysis demonstrates that reduced ITPase activity is associated with reduced plasma RBV levels, which in turn is also associated with a greater protection against RBV-induced anemia.

Previous reports demonstrated an association between reduced ITPase activity and treatment-induced thrombocytopenia [29,45], likely in relation to the decline in Hb, or due to the increased stimulation of megakaryocyte-erythroid progenitor cells by erythropoietin production [46]. Our data also demonstrate a larger change in PLT count with lower ITPase function, a change that leads to higher PLT counts in the DAA+ RBV arm. Interestingly, this is the opposite to what was previously noted in IFN-containing HCV therapy, e.g. the NORDynamIC trial[29]. We hypothesize that this difference is likely due to the absence of IFN in our trial. Interestingly, we also see a trend towards a larger decrease in PLT counts with increasing ITPase function in the placebo for RBV-containing arm which may be unrelated to the erythrocyte-mediated effect of ITPase function.

Our work broadens our understanding of the important role genetics plays in HCV treatment response, regardless of whether the therapy is IFN-based or IFN-free. Recent studies have outlined the impact of host pharmacogenetics as predictors of HCV treatment outcome, especially given the variability of treatment responses in different populations.[47] By adding multiple genetic variants to the model, there are more predictive capabilities that can be learned. For instance, a recent paper identified a ‘formula’ using available metadata which could predict with high accuracy, the chances of SVR without hemolysis and toxicity. This was based on the genotypes at three genes of interest IL28B, ITPA, and SLC28A3 [48]. Genetics-based personalized anti-HCV therapy may well be a future approach of choice, especially within populations with harder to cure HCV infections, e.g. decompensated cirrhosis, which likely will continue to require the continued use of ribavirin.

## Supporting information

S1 Fig Hb change least square means by treatment arm. (PDF)

S2 Fig Hb change least square means by rs12979860 genotype. (PDF)

S3 Fig Hb change as a function of sex. (PDF)

S4 Fig Log2Ctrough least square means by rs12979860 genotype. (PDF)

S5 Fig Change in PLT counts as a function of rs12979860 genotype. (PDF)

S6 Fig Estimated change in PLT counts by an interaction of treatment arm and rs12979860 genotype. (PDF)

S1 Table List of IRB sites in which pharmacogenetic analysis was approved. (PDF)

S2 Table PCR, pyrosequencing primers used for IL28b rs12979860 and ITPA genetic analyses. (PDF)

S3 Table Hardy-Weinberg equilibrium test results for bialleleic ITPA SNPs rs7270101 and rs1127354 as calculated using Santiago Rodriguez, Tom R. Gaunt, and Ian N.M. Day 2009. (PDF)

S4 Table Mean Hb (g/dL) and mean PLT (K/μL) at baseline and end of treatment stratified by ITPase functional activity and treatment arm (+RBV/Placebo for RBV). (PDF)

S5 Table Response to the 3-DAA regimen by measuring sustained virological response at 12 weeks post-treatment (SVR12). (PDF)
