# Supplementary material for: Reduced ITPase activity and favorable IL28B genetic variant protect against ribavirin-induced anemia in interferon-free regimens
Source: PLoS One. 2018 May 31;13(5):e0198296. doi: 10.1371/journal.pone.0198296 (PMC5979032; doi:10.1371/journal.pone.0198296)
Supplement: S1 Fig — (PDF) [file pone.0198296.s001.pdf]

S1 Fig. Hb Change Least Square Means by Treatment Arm

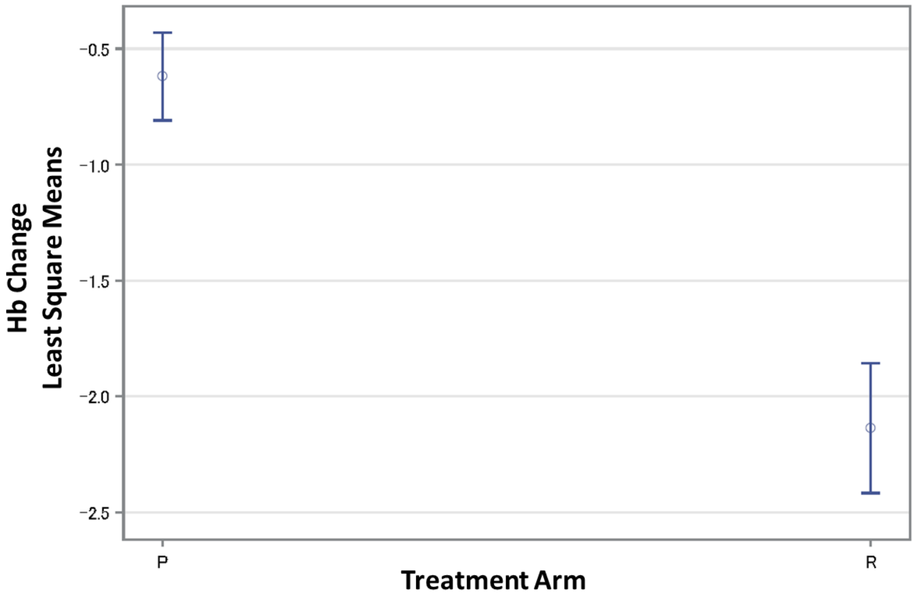

S1 Fig. The least square means were calculated for Hb change using age, gender, and *rs12979860* genotype and ITPase functional activity for the 2 arms. Circles indicate the LS means, error bars indicate the 95% confidence interval of the LS-means. P= DAA + placebo; R= DAA + Ribavirin

Hb Change LSMean for Placebo Arm (P)= -0.6189 (SE=0.0961)

Hb Change LSMean for Ribavirin Arm (R)=-2.136 (SE=0.1417)

There was a significant drop in Hb levels in RBV-containing arm (P<0.001)
