# Supplementary material for: Reduced ITPase activity and favorable IL28B genetic variant protect against ribavirin-induced anemia in interferon-free regimens
Source: PLoS One. 2018 May 31;13(5):e0198296. doi: 10.1371/journal.pone.0198296 (PMC5979032; doi:10.1371/journal.pone.0198296)
Supplement: S2 Fig — (PDF) [file pone.0198296.s002.pdf]

S2 Fig. Hb Change Least Square means by rs12979860 Genotype

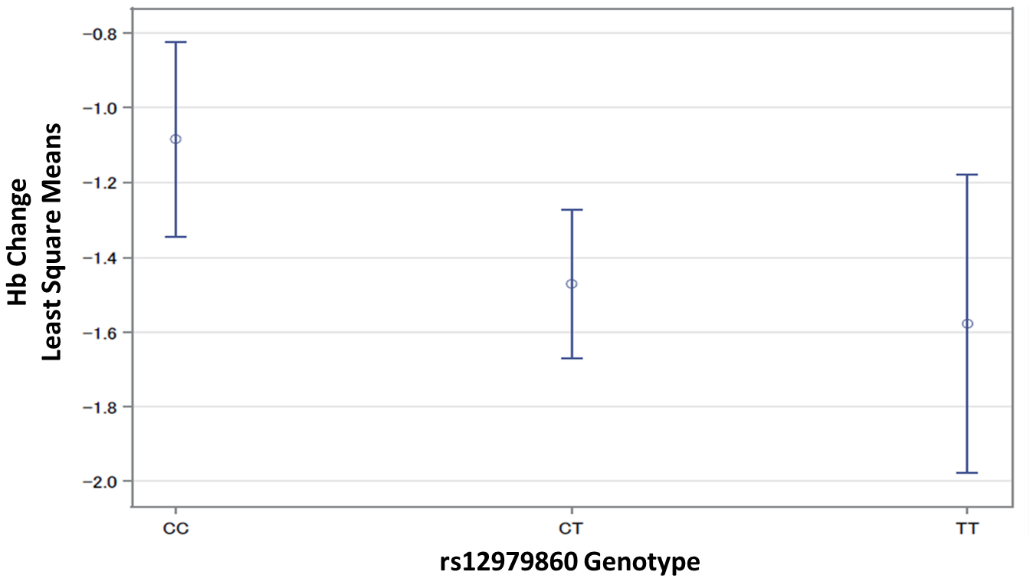

S2 Fig. The least square means were calculated for Hb change using age, sex, arm, ITPase function for the *rs12979860* genotype. Circles indicate the LS means, error bars indicate the 95% confidence interval of the LS-means. We found a larger, significant reduction (P=0.0367) in Hb when there was at least one 'T' allele present.
