# Supplementary material for: Reduced ITPase activity and favorable IL28B genetic variant protect against ribavirin-induced anemia in interferon-free regimens
Source: PLoS One. 2018 May 31;13(5):e0198296. doi: 10.1371/journal.pone.0198296 (PMC5979032; doi:10.1371/journal.pone.0198296)
Supplement: S3 Fig — (PDF) [file pone.0198296.s003.pdf]

S3 Fig. Hb Change as a Function of Sex

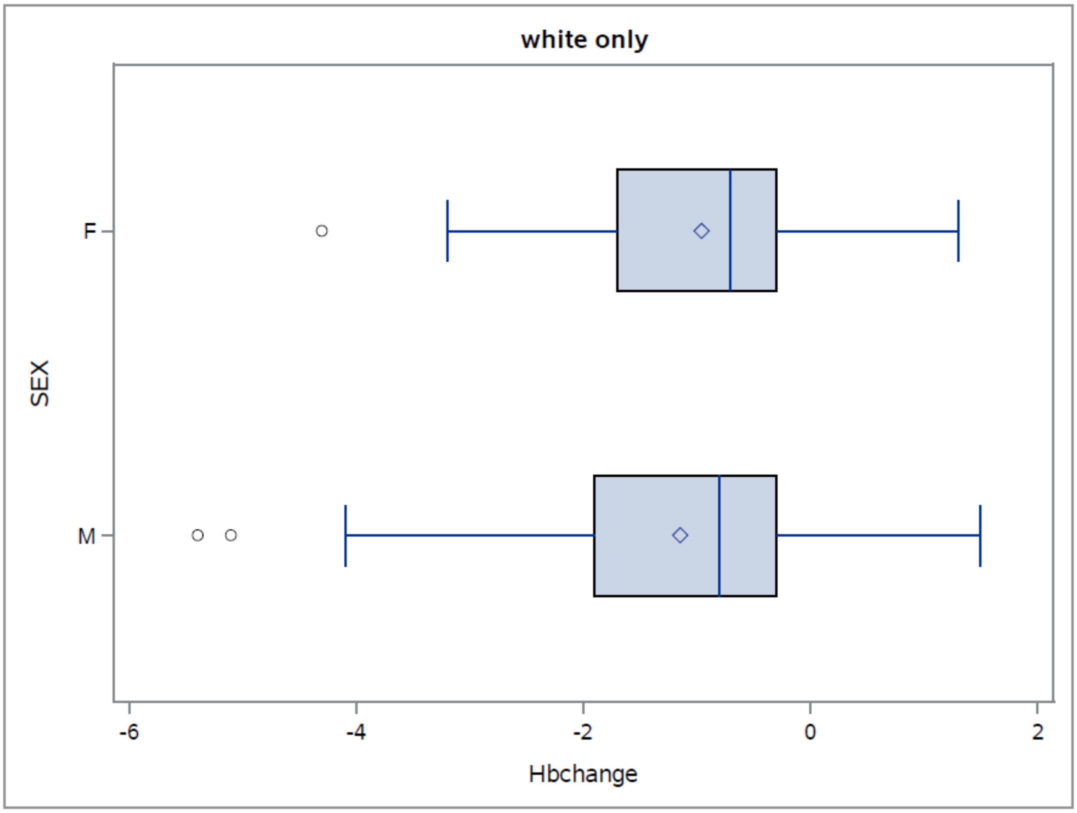

S3 Fig. Box and whiskers plots show means and 95% Confidence intervals. No observable significant differences in Hb changes between male (M) and female (F) patients.
