# Supplementary material for: Reduced ITPase activity and favorable IL28B genetic variant protect against ribavirin-induced anemia in interferon-free regimens
Source: PLoS One. 2018 May 31;13(5):e0198296. doi: 10.1371/journal.pone.0198296 (PMC5979032; doi:10.1371/journal.pone.0198296)
Supplement: S5 Fig — (PDF) [file pone.0198296.s005.pdf]

S5 Fig. Change in PLT counts as a function of rs12979860 genotype

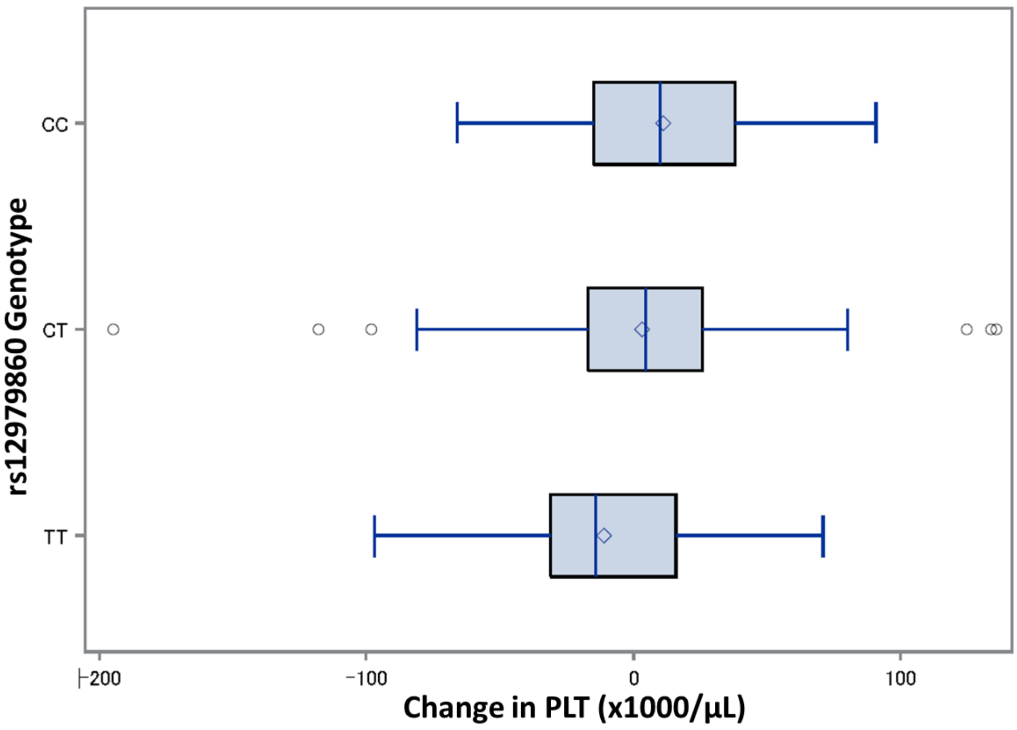

S5 Fig. Overall shifts in PLT change plotted out as a function of rs12979860 genotype. Box and whiskers plots show means and 95% Confidence intervals. We did not observe significant differences in PLT changes between subjects carrying CC, CT, and TT genotypes at theIL-28b locus.
