# Supplementary material for: Reduced ITPase activity and favorable IL28B genetic variant protect against ribavirin-induced anemia in interferon-free regimens
Source: PLoS One. 2018 May 31;13(5):e0198296. doi: 10.1371/journal.pone.0198296 (PMC5979032; doi:10.1371/journal.pone.0198296)
Supplement: S6 Fig — (PDF) [file pone.0198296.s006.pdf]

S6 Fig. Estimated Change in PLT counts by an Interaction of treatment arm and rs12979860 genotype

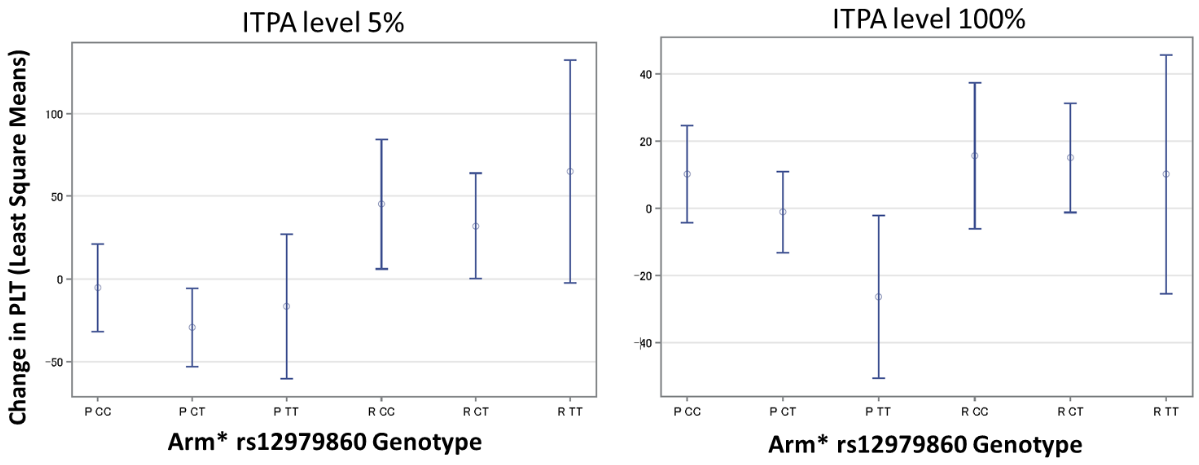

S6 Fig. The least square means were calculated for platelet change from baseline to EOT using age and sex as covariates. Circles indicate the LS means, error bars indicate the 95% confidence interval of the LS-means. At the ITPase functional levels 5% (A) and 100% (B), there are significant differences between arms, but no significant association with *rs12979860* genotype. P=Placebo treatment arm, R=RBV treatment arm.
