# Supplementary material for: Reduced ITPase activity and favorable IL28B genetic variant protect against ribavirin-induced anemia in interferon-free regimens
Source: PLoS One. 2018 May 31;13(5):e0198296. doi: 10.1371/journal.pone.0198296 (PMC5979032; doi:10.1371/journal.pone.0198296)
Supplement: S1 Table — (PDF) [file pone.0198296.s007.pdf]

S1 Table. List of IRB sites in which pharmacogenetic analysis was approved

#	Site Name	Approving IRB/IEC
1	Queen Alexandra Hospital, Portsmouth, UK	NRES committee London City and East, Bristol, UK
2	Medical Associates Research Group, San Diego, CA, USA	Quorum Review IRB, Seattle WA USA
3	North Shore- Long Island Jewish Health System, Manhasset, NY, USA	Biomedical research Alliance of New York, Lake Success, NY, USA
4	Delta Research Partners, Bastrop, LA, USA	Quorum Review IRB, Seattle WA USA
5	Clinical Research Centers of America, Murray, UT, USA	Quorum Review IRB, Seattle WA USA
6	Metropolis medical group San Francisco CA, USA	Quorum Review IRB, Seattle WA USA
7	St Marys Hospital, London, UK	NRES committee London –London Bridge, London, UK
8	Ottawa Hospital, Ottawa ON, Canada	Ottawa Hospital Research Ethics board, Ottawa, ON, Canada
9	Clinique Medicale du Quartier Latin, Montreal, QC, Canada	Quorum Review IRB, Seattle WA USA
10	Derriford Hospital, Plymouth, UK	NRES committee London City and East, Bristol, UK
11	South Florida Center of Gastroenterology, Wellington, FL, USA	Quorum Review IRB, Seattle WA USA
12	The Royal Free Hospital, London, UK	NRES committee London City and East, Bristol, UK
13	Florida Medical Clinic Zephyrhills, FL, USA	Quorum Review IRB, Seattle WA USA
14	Toronto Liver Center, Toronto, ON, Canada	Quorum Review IRB, Seattle WA USA
15	Kaiser Permanente, San Francisco, CA, USA	Kaiser Permanente Northern California IRB, Oakland, CA, USA
16	St. George’s Hospital, London, UK	NRES committee London City and East, Bristol, UK
17	Chelsea and Westminster Hospital, London, UK	NRES committee London –London Bridge, London, UK
18	Kansas City research Institute, Kansas City, MO, USA	Quorum Review IRB, Seattle WA USA
19	Cure C Consortium, Houston, TX, USA	Quorum Review IRB, Seattle WA USA
20	Wisconsin Center for Advanced Research, Milwaukee, WI, USA	Quorum Review IRB, Seattle WA USA
21	eStudySite, La Mesa, CA, USA	Quorum Review IRB, Seattle WA USA
22	The Ohio State University Wexner, Columbus, OH, USA	Western Institutional Review board, Olympia, WA, USA
23	Charlotte Gastroenterology and hepatology, Charlotte, NC, USA	Quorum Review IRB, Seattle WA USA
24	Southern California Research Center, Coronado, CA, USA	Quorum Review IRB, Seattle WA USA
25	Quality medical Research, Nashville, TN,	Quorum Review IRB, Seattle WA USA

	USA	
26	Inland Empire Liver Foundation, Rialto CA, USA	Quorum Review IRB, Seattle WA USA
27	Tampa General Medical Group, Tampa General Hospital, Tampa, FL< USA	Quorum Review IRB, Seattle WA USA
28	Trial management Assocaites, Wilmington, NC, USA	Quorum Review IRB, Seattle WA USA
29	Quest Clinical research, San Francisco, CA, USA	Quorum Review IRB, Seattle WA USA
30	Clinique medicale L'Actuel, Montreal, QC, Canada	Quorum Review IRB, Seattle WA USA
31	eStudySite, Oceanside, CA, USA	Quorum Review IRB, Seattle WA USA
32	eStudySite, San Diego, CA, USA	Quorum Review IRB, Seattle WA USA
33	ID Care of New jersey, Hillsborough, NJ, USA	Quorum Review IRB, Seattle WA USA
34	Tampa General medical Group, Tampa General Hospital, Tampa, FL, USA	Quorum Review IRB, Seattle WA USA
35	Midland Florida Clinical Research, DeLand, FL, USA	Quorum Review IRB, Seattle WA USA
36	Triple O Research Institute, West Palm Beach, FL, USA	Quorum Review IRB, Seattle WA USA
37	eStudySite, Chula Vista, CA, USA	Quorum Review IRB, Seattle WA USA
38	Gartnavel General Hospital, Glasgow, Scotland, US	NRES committee London City and East, Bristol, UK
39	Midway Immunology and Research Center, Fort Pierce, FL, USA	Quorum Review IRB, Seattle WA USA
40	GIRI Gastrointestinal Research Institute, Vancouver, BC, Canada	Quorum Review IRB, Seattle WA USA
41	Atlanta Center for Gastroenterology PC, Decatur, GA, USA	Quorum Review IRB, Seattle WA USA
42	Consultants for Clinical Research, Cincinnati, OH, USA	Quorum Review IRB, Seattle WA USA
43	Abbott Northwestern Hospital, Minneapolis, MN, USA	Quorum Review IRB, Seattle WA USA
44	Orange Coast Medical Group, Newport beach, CA, USA	Quorum Review IRB, Seattle WA USA
45	Peter J Ruane, MD, Inc., Los Angeles, CA, USA	Quorum Review IRB, Seattle WA USA
46	Metropolitan Research, Annandale, VA, USA	Quorum Review IRB, Seattle WA USA
47	University Hospital Queen's Medical Centre, Nottingham, UK	NRES committee London City and East, Bristol, UK
48	University of Alabama at Birmingham, Center for AIDS Research UAB, Birmingham, AL, USA	Western Institutional Review Board, Olympia, WA, USA
49	Aga Clinical Research Associates, LLC, Egg Harbor Township, NJ, USA	Quorum Review IRB, Seattle WA USA

50	University Gastroenterology, providence RI, USA	Quorum Review IRB, Seattle WA USA
51	LAIR Centre, Vancouver, BC, Canada	Quorum Review IRB, Seattle WA USA
52	The Queens medical Center, Honolulu, HI, USA	The Queens medical Center Institutional and Research Review Committee, Honolulu, HI, USA
53	UCSD Antiviral Research Center, San Diego, CA, USA	University of California- San Diego, Human research protections Program, La Jolla, CA, USA
54	Gastro One, Germantown, TN, USA	Quorum Review IRB, Seattle WA USA
55	Inova Fairfax Hospital Center for Liver Diseases, Falls Church, VA, USA	Quorum Review IRB, Seattle WA USA
56	Lehigh Valley Health network/Lehigh Valley Hospital Allentown, PA, USA	Lehigh Valley Health Network/Lehigh Valley Hospital Institutional Review Board Research Participant Protection Office, Allentown, PA, USA
