# Supplementary material for: Reduced ITPase activity and favorable IL28B genetic variant protect against ribavirin-induced anemia in interferon-free regimens
Source: PLoS One. 2018 May 31;13(5):e0198296. doi: 10.1371/journal.pone.0198296 (PMC5979032; doi:10.1371/journal.pone.0198296)
Supplement: S2 Table — (PDF) [file pone.0198296.s008.pdf]

S2 Table. PCR, pyrosequencing primers used for *IL28b rs12979860* and *ITPA* genetic analyses

Primer Name	Primer Sequence	Amplicon Length
rs12979860_Fwd	TTGCTGGGGGAGCGCGGAG	**291**
rs12979860_RevBio	/5Biosg/TGCCTCTCCCGCCCACTCCC	
rs12979860_Seq	GCAATTCAACCCTGGTTC	
ITPA_rs1127354_FwdBio	/5Biosg/TGG AAC AGG TCG TTC AGA TTC TA	**219**
*ITPA*_rs1127354_Rev	CGA ACT GCC TCC TGA CAT TT	
*ITPA*_rs1127354_Seq	GCC ACC AAA GTG CAT	
*ITPA*_rs7270101_FwdBio	/5Biosg/TGG AAC AGG TCG TTC AGA TTC TA	**219**
*ITPA*_rs7270101_Rev	CGA ACT GCC TCC TGA CAT TT	
*ITPA*_rs7270101_Seq	AAA TCC AAC CAT CTT TTA A	
