# Supplementary material for: Reduced ITPase activity and favorable IL28B genetic variant protect against ribavirin-induced anemia in interferon-free regimens
Source: PLoS One. 2018 May 31;13(5):e0198296. doi: 10.1371/journal.pone.0198296 (PMC5979032; doi:10.1371/journal.pone.0198296)
Supplement: S3 Table — (PDF) [file pone.0198296.s009.pdf]

S3 Table. Hardy-Weinberg Equilibrium Test Results for Bialleleic *ITPA* SNPs *rs7270101* and *rs1127354* as Calculated Using Santiago Rodriguez, Tom R. Gaunt, and Ian N.M. Day 2009

SNP Tested	Genotype	Expected	Observed	p allele Frequency
**rs7270101**	T/T	169.35	168	0.90
	T/G	54.3	57	0.88
	G/G	4.35	3	0.85
**rs1127354**	G/G	188.93	189	0.90
	G/T	38.15	38	0.91
	T/T	1.93	2	0.91
