# Supplementary material for: Reduced ITPase activity and favorable IL28B genetic variant protect against ribavirin-induced anemia in interferon-free regimens
Source: PLoS One. 2018 May 31;13(5):e0198296. doi: 10.1371/journal.pone.0198296 (PMC5979032; doi:10.1371/journal.pone.0198296)
Supplement: S4 Table — (PDF) [file pone.0198296.s010.pdf]

S4 Table. Mean Hb (g/dL) and Mean PLT (K/μL) at Baseline and End of Treatment Stratified by ITPase Functional Activity and Treatment Arm (+RBV/Placebo for RBV)

ITPase functional activity	Arm	Number	Mean Hb at Baseline ± SD (g/dL)	Mean Hb at EOT ± SD (g/dL)	Mean PLT at Baseline ± SD (K/μL)	Mean PLT at EOT ± SD (K/μL)
100 %	+RBV	41	14.59 ± 1.28	12.22 ± 1.21	233.5 ± 56.65	249.3 ± 56.65
	Placebo	93	14.51 ± 1.43	13.98 ± 1.44	235.8 ± 65.58	234.5 ± 67.72
60%	+RBV	14	15.12 ± 1.53	13.15 ± 1.92	210.0 ± 33.29	241.9 ± 52.36
	Placebo	36	14.93 ± 1.22	14.20 ± 1.25	220.7 ± 55.26	209.4 ± 51.18
25-30%	+RBV	12	14.18 ± 1.40	13.35 ± 1.39	213.1 ± 53.14	248.3 ± 74.64
	Placebo	20	15.49 ± 1.51	14.67 ± 1.40	230.3 ± 44.14	227.6 ± 46.54
≤10%	+RBV	1	15.00	12.9	237.0	209.0
	Placebo	7	15.06 ± 1.47	14.69 ± 1.21	242.0 ± 112.3	234.7 ± 96.47
