# Supplementary material for: Reduced ITPase activity and favorable IL28B genetic variant protect against ribavirin-induced anemia in interferon-free regimens
Source: PLoS One. 2018 May 31;13(5):e0198296. doi: 10.1371/journal.pone.0198296 (PMC5979032; doi:10.1371/journal.pone.0198296)
Supplement: S5 Table — (PDF) [file pone.0198296.s011.pdf]

S5 Table. Response to the 3-DAA Regimen by Measuring Sustained Virological Response at 12 Weeks Post-Treatment (SVR12)

ITPase functional activity	# Patients per Arm		Number of Patients Achieving SVR12	
	**-RBV**	**+RBV**	**-RBV**	**+RBV**
100 %	93	41	7	0
60%	36	14	0	0
25-30%	20	12	2	0
≤10%	7	1	0	0
